# Cost and operational impact of promoting upfront GeneXpert MTB/RIF test referrals for presumptive pediatric tuberculosis patients in India

**DOI:** 10.1371/journal.pone.0214675

**Published:** 2019-04-01

**Authors:** Sanjay Sarin, Sophie Huddart, Neeraj Raizada, Debadutta Parija, Aakshi Kalra, Raghuram Rao, Virender Singh Salhotra, Sunil D. Khaparde, Catharina Boehme, Claudia M. Denkinger, Hojoon Sohn

**Affiliations:** 1 Foundation for Innovative New Diagnostics, New Delhi, India; 2 Department of Epidemiology, Biostatistics and Occupational Health, McGill University, Montreal, Quebec, Canada; 3 McGill International TB Centre, Montreal, Quebec, Canada; 4 Central TB Division, Government of India, New Delhi, India; 5 Foundation for Innovative New Diagnostics, Geneva, Switzerland; 6 Johns Hopkins Bloomberg School of Public Health, Baltimore, Maryland, United States of America; Jamia Hamdard, INDIA

## Abstract

**Background:**

Outreach and promotion programs are essential to ensuring uptake of new public health interventions and guidelines. We assessed the costs and operation dynamics of outreach and promotion efforts for up front Xpert MTB/RIF (Xpert) testing for pediatric presumptive tuberculosis (TB) patients in four major Indian cities.

**Methods:**

Xpert test costs were assessed as weighted average per-test costs based on the daily workload dynamics matched by test volume specific Xpert unit cost at each study site. Costs of outreach programs to recruit health providers to refer pediatric patients for Xpert testing were assessed as cost per referral for each quarter based on total program costs and referral data. All costs were assessed in the health service provider’s perspective and expressed in 2015 USD.

**Results:**

Weighted average per-test costs ranged from $14.71 to $17.81 at the four laboratories assessed. Differences between laboratories were associated with unused testing capacity and/or frequencies of overtime work to cope with increasing demand and same-day testing requirements. Outreach activities generated between 825 and 2,065 Xpert testing referrals on average each quarter across the four study sites, translating into $0.63 to $2.55 per patient referred. Overall outreach costs per referral decreased with time, stabilizing at an average cost of $1.10, and demonstrated a clear association with increased referrals.

**Conclusions:**

Xpert test and outreach program costs within and across study sites were mainly driven by the dynamics of Xpert testing demand resulting from the combined outreach activities. However, these increases in demand required considerable overtime work resulting in additional costs and operational challenges at the study laboratories. Therefore, careful laboratory operational adjustment should be evaluated at target areas in parallel to the anticipated demand from the Xpert referral outreach program scale-up in other Indian regions.

## Introduction

In 2016, India accounted for more than one quarter of world’s reported TB cases and deaths despite a dramatic 37% improvement in case notification rate between 2013 and 2016. Globally, children (aged <15 years) accounted for 6.9% of the notified new TB cases in 2016 [1]. Given that prompt diagnosis and linkage to care are integral components of TB control, the Indian government has made it a priority to expand rapid molecular diagnostic capacity for TB using Xpert MTB/RIF (referred to as Xpert from here on; Cepheid, Sunnyvale) test as part of India’s plan to eliminate TB by 2025 [2]. However, scale-up of Xpert testing capacity and coverage alone does not ensure that all patients who could benefit from Xpert are referred for testing. Education and outreach activities to make health providers aware of the need and local testing availabilities are thus necessary to ensure uptake of Xpert testing.

Between April 2014 and June 2016, the Foundation for Innovative New Diagnostics (FIND), in collaboration with the Revised National Tuberculosis Control Program (RNTCP), conducted a large-scale project to catalyze the adoption of upfront Xpert testing in presumptive pediatric TB cases as a routine clinical practice utilizing multiple outreach interventions–phone calls, one-on-one provider sensitization meetings, and Continuing Medical Education (CME) workshops for public and private sector health providers. This project was undertaken in four major cities of India: New Delhi, Chennai, Hyderabad and Kolkata with a total of 3,670 health providers educated and informed of the upfront Xpert testing strategy for presumptive pediatric TB patients [3]. The phone calls and one-on-one meetings with health providers included descriptions of project deliverables and mandates as well as informing the health providers of the availability of testing facilities and relevant patient referral procedures. CME workshops further included presentations by key speakers providing the details of the World Health Organization (WHO) guidelines on the use of Xpert for treatment of TB patients and promoting referrals of presumptive pediatric TB patients for Xpert testing.

These demand-generating activities were an integral component of the program and helped to increase access to testing and optimize efficiency of testing [3]. During the study period, the program provided 46,879 GeneXpert tests to 42,238 children identifying 3,340 TB cases. More than 92% of the patients received test results within 24 hours of sample receipt at the lab. This program is now fully managed by the RNTCP and has been expanded to an additional six cities as of 2017 –Surat, Visakhapatnam, Guwahati, Bangalore, Nagpur and Indore–with plans to further scale-up to other cities in India.

Given the positive impact of the FIND/RNTCP pediatric Xpert implementation project and the RNTCP’s commitment to expand the combined outreach program alongside of increasing Xpert testing coverage, it is important to evaluate the cost implications of the combined outreach efforts and their impact on laboratory operations and workloads. We used the FIND/RNTCP pediatric Xpert implementation project data to provide empiric evidence on costs of outreach program operations, and their impact on cost and operation of the Xpert testing at four project laboratories.

## Methods

The objective of our work was to assess the economic costs of the FIND/RNTC outreach program in promoting upfront Xpert testing for pediatric presumptive TB patients and respective laboratory testing costs at the four study locations from the health systems perspective. As such, we 1) evaluated costs of the three outreach programs, 2) evaluated the cost dynamics of Xpert tests associated with changes in the workload observed at four laboratories during the study period; and 3) explored how outreach efforts influenced the demand for Xpert referrals for pediatric TB diagnosis.

### FIND/RNTCP pediatric Xpert implementation project

Data were collected from a USAID-funded implementation project of upfront GeneXpert MTB/RIF testing of presumptive pediatric TB patients [3]. The project enrolled pediatric (age 0–14 years) presumptive TB and Drug-Resistant (DR) TB patients in the catchment areas of the four RNTCP reference level laboratories in New Delhi, Chennai, Hyderabad and Kolkata. Patients provided various types of specimen (e.g. induced sputum, gastric lavage/aspirate, BAL, CSF, lymph node aspirates, etc.) for Xpert testing. Test results were promptly communicated to the health providers by e-mail and short messaging service (SMS).

### Evaluation of Xpert per-test unit cost

For New Delhi, Hyderabad and Kolkata laboratories, we first calculated a reference table of Xpert per-test costs relevant to sample batch sizes ranging between 1 and 16 tests using bottom-up activity-based costing (ABC) framework [4]. For Chennai, overhead costs were difficult to obtain; thus, we used the New Delhi lab costs based on its similarity in size and capacity. Data on all resource inputs required for Xpert testing was obtained from Time and Motion (TAM) studies [5] conducted as part of previous FIND cost and cost-effectiveness studies of Xpert in India, Vietnam, and Malawi. The TAM data was collected based on repeated direct observations of sample batch sizes between 1 to 16 tests that typically covers a range of daily workload capacity using a single GeneXpert IV (GX4) machine. We opted to use the existing TAM data instead of conducting a new TAM as Xpert testing procedure is simple and highly standardized such that operator variability would not be a significant factor influencing the unit cost of an Xpert test. A resource-use and overhead cost questionnaire was utilized to reflect site specific costs, resource use and unit prices.

Resource inputs were categorized as overhead, building, equipment, staff, laboratory reagents and chemicals, and consumables. Unit price of commodities (e.g. laboratory reagents, chemical, consumables), staff salaries, and overhead cost data were obtained from each laboratory’s relevant accounting and inventory management records for the 2015 fiscal year. As government operated laboratories do not pay for building and utility related costs, these data were supplemented from a similar private non-profit laboratory in India offering Xpert test [6]. Use and costs of capital assets were based on annualized costs using a 3% discount rate [7,8] and assumed expected life-years as specified in FIND’s internal documents [9]. All costs were evaluated and expressed in 2015 United States Dollars (USD) where cost data items in local currencies were converted to the USD according to the average UN operational exchange rate in 2015 [10].

### Costs associated with unused GeneXpert equipment capacity

As underused Xpert testing capacity is an important opportunity cost associated with the implementation of Xpert testing, we sought to incorporate the costs of unused Xpert testing capacity as part of the per-test unit costs. In the case of Xpert testing, the type of GeneXpert testing platform likely determines the maximum daily testing capacity in a fixed laboratory operating time. The four study sites used GX4 units, which were equipped with four testing modules (each slot as able to hold one Xpert cartridge). Given that each Xpert test takes around 2 hours to complete, a single GX4 machine would have a daily maximum capacity to process 16 tests in a laboratory that normally operates between 8 and 10 hours each day. Any number of tests performed less than 16 in each operational day would result in incorporation of costs associated with unused GX4 module(s). For example, if a laboratory performed 10 Xpert tests in a given day, we added costs equivalent to 6 individual GX4 module capacities as part of the total testing costs for that day.

### Workload analysis and weighted per-test cost

Using the daily workload data at each laboratory, we assigned a reference per-test cost to each operational day and then calculated the weighted average per-test cost of an Xpert test unique for each study site. Since some laboratories had multiple GX4 units and had workload requiring overtime operations, we assumed sample batching would be cost-optimized for GX4 utilization (i.e. a combination that would have lowest cost associated with unused GX4 capacity). For example, if 28 tests were run on a given day, we determined three sample batching of one 16-test, one 10-test batch, and one 2-test batch would be cost-optimized. This process was repeated for each laboratory using laboratory specific reference per-test cost and averaged over the length of the experiment to obtain the average weighted per-test Xpert cost. A sample calculation can be viewed in S1 Calculation.

To ensure 24-hour turn-around of Xpert test results, some laboratories were equipped with more than one GX4 machines and operated overtime when workloads were beyond their normal testing capacity. To assess the burden of overtime work, we calculated the proportion of days with workloads exceeding each laboratory’s normal testing capacity (determined based on the number of GX4 machines available and a standard 8 hour work day). Costs associated with overtime work were then assessed based on the total estimated hours of overtime multiplied by hourly staff wages and laboratory overhead costs. We assumed each incremental batch of 4 tests (1 to 4 tests) performed beyond normal testing capacity took at least two hours of operational time to run.

### Evaluation of outreach spending

Any health provider in the four cities with presumptive pediatric TB patients was eligible to refer patients for free pert testing. To promote referrals for Xpert testing during the study, our trained project staff placed phone calls, held one-on-one meetings and conducted CME workshops in the four cities.

Health providers catering to the healthcare needs of the pediatric population were mapped and were systematically approached first by telephone. Telephone contact was followed-up, where feasible, by a one-on-one meeting between the health provider and a member of the project team. These efforts were complemented by periodic comprehensive CME events. During telephone calls and one-on-one meetings, representatives of the project explained the Xpert test and how to refer presumptive pediatric TB patients for a free upfront Xpert test for bacteriologic diagnosis. CME events also covered topics relevant to the diagnosis and treatment of pediatric TB.

Cost data on each of the outreach efforts included staff wages, estimated staff and activity time associated with planning and carrying out each of the outreach activities, expenditure data on consumable, per-diem, phone call charges, travel related costs (for CMEs and one-on-one visits), and time required for management and supervision. Regarding transportation costs, staff often visited multiple providers or locations per trip and used public transportation. However, detailed records were not kept, so our costing assumes one CME or meeting per trip and a standard distance of 10km travelled using a taxi.

Data on the telephone calls and one-on-one meetings (e.g. number calls or visits made, duration, expenses) were collected for a representative monthly sample of calls and visits made during the study period. Data on CME were obtained through retrospective review of all CME meeting and relevant FIND financial records.

We estimated the unit costs of each outreach activity using a top-down cost analysis method where the total expenditure on consumables, staff time, and transportation for a given recruitment activity was divided by the frequency of each activity conducted during the study period to compute the average unit costs. Overhead costs were calculated as 15% of operational costs as per FIND’s overhead pricing structure. To express uncertainties in our cost estimates, we constructed high and low-cost estimates based on varied levels of staff and transportation costs (+/- 20%) and percent allotment for overhead (ranges: 10 to 20%) respectively.

Data entry and analyses were performed using Excel 2016 (Microsoft Corporation) and R (version 3.5.0) for workload analyses.

### Funding

The FIND/RNTCP pediatric GeneXpert implementation project was funded by United States Agency for International Development (USAID) under the Challenge TB project. FIND was responsible for the implementation, training, coordination, monitoring, data analysis and writing of the report in close coordination with the Central TB Division, Ministry of Health and Family Welfare, Government of India.

### Ethics

Xpert testing for pediatric presumptive TB cases is an approved intervention under the RNTCP. The current project was undertaken by FIND, after approval from and in collaboration with RNTCP. As such, the results presented here are our experience-sharing of implementing approved interventions in a programmatic setting within the existing accredited RNTCP TB diagnostic lab network. Since the observations described here are a part of implementation of approved interventions under the RNTCP and a part of standard TB care in India, separate ethical clearance was not required. The only patient data used for this work was the date of GeneXpert testing and this data was fully anonymized before being shared for analysis.

## Results

### Xpert cost and laboratory operations

**Fig 1:**
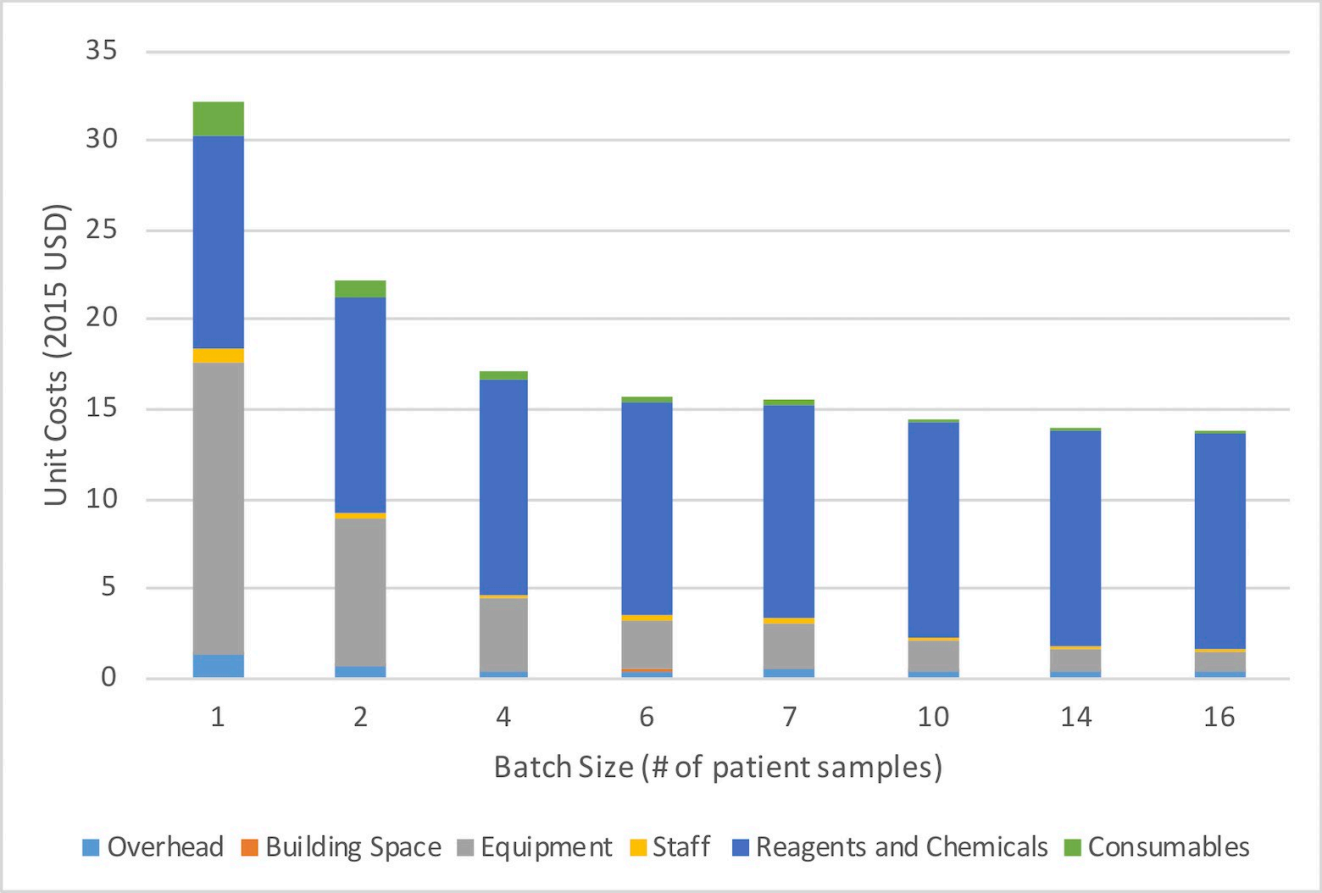
Reference Xpert per test costs for different testing workloads (batch size) categorized by type of resource inputs (New Delhi Tuberculosis Centre). Overhead includes electricity, gas, water, cleaning services etc. as well as staff involved in running the laboratories who were not employed by the FIND/RNTCP pediatric Xpert implementation project. Building space includes rent for rooms used for Xpert testing. Equipment includes the Xpert machine and other lab equipment. Staff includes wages for lab and managerial staff. Regents and chemicals consists of Xpert cartridge costs. Consumables include lab gloves and other personal protective equipment.

**Fig 2:**
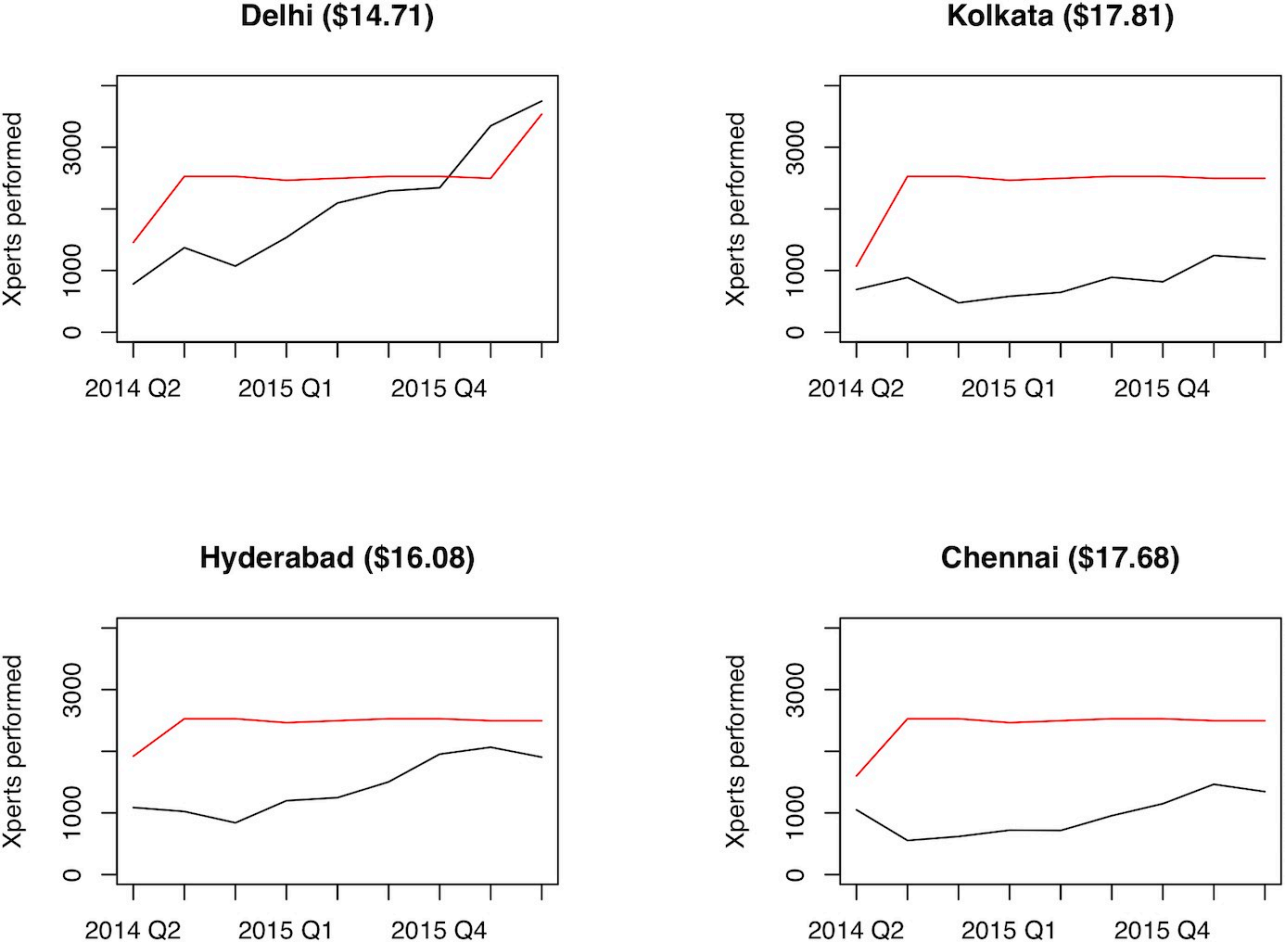
Quarterly workload data by city. Black indicates quarterly workload and red indicates quarterly testing capacity based on standard operating hours. Values in parentheses are the average workload-weighted Xpert per-test unit costs.

Reference per-test costs ranged from $13.74 to $32.14 in New Delhi (Fig 1, S1 Table) with the lowest cost associated with a batch size of 16. In Kolkata, unit costs ranged from $13.55 to $30.23 and from $13.74 to $30.85 in Hyderabad (S1 Table). We were not able to evaluate overhead costs in Chennai due to the complexity of its operation (a mix of routine clinical and research tasks) making it difficult to disintegrate overhead cost for routine Xpert testing only. The major cost drivers were equipment costs and reagents (mainly the cost of the GeneXpert machine–decreasing with increasing batch size–and the Xpert cartridge) while overhead and direct staff hands-on cost had minimal impact. The weighted average per-test cost for each laboratory during this experiment ranged from $14.71 to $17.81, depending on the demand of Xpert test observed in this experiment (Table 1 and Fig 2).

**Table 1:** Total per-test unit costs during the experiment, weighted to account for actual different unit costs by workloads, and costs associated with overtime work and laboratory operations to meet same-day testing.

City	Total testing costs	Weighted avg. per-test cost	Total overtime days (%)	Workweek overtime days (%)	Sunday overtime days (%)	Overtime testing costs (staff and building costs)
New Delhi	$342,115.10	$14.71	179 (25.4%)	173 (21.1%)	6 (0.7%)	$47,440.38 ($769.14)
Kolkata	$147,790.70	$17.81	37 (5.3%)	28 (3.4%)	9 (1.1%)	$4,565.22 ($106.18)
Hyderabad	$243,347.80	$16.08	114 (16.2%)	70 (8.5%)	44 (5.4%)	$20,228.40 ($403.36)
Chennai	$168,121.90	$17.68	29 (4.1%)	29 (4.1%)	0 (0.0%)	$5,007.55 ($124.10)

Overall, the demand for Xpert testing increased during the study period, particularly following the CME efforts. In New Delhi, it resulted in testing demand consistently exceeding the laboratory capacity (Fig 2). Consequently, overtime work was most significant at the New Delhi laboratory, primarily at the later stages of the experiment, with 25.4% (179 days, inclusive of Sunday operations) of the 821 days in the study period recording overtime work, followed by 16.2% (114 days) in Hyderabad, 5.3% (37 days) in Kolkata and 4.1% (29 days) in Chennai (Table 1). In New Delhi, Kolkata and Chennai overtime generally occurred as extended operating hours during the workweek, Monday through Saturday. In Hyderabad, 44 of the 114 overtime days were Sundays when the lab was scheduled to be closed. The majority of the costs associated with overtime testing were associated with theXpert cartridge and other consumables. Total costs associated with overtime staff and building costs were less than $1,000 in each city.

### Outreach and recruitment

A complete cost summary of outreach activities is presented in Table 2. Unit costs for each type of outreach activity was $0.16 (range $0.12 to $0.20) per phone call, $7.14 (range $5.92 to $9.23) per one-on-one meetings with health providers, and $7.88 (range $6.03 to $9.86) per attendee at a CME. A total of 33 CMEs were held across the four cities with an average attendance of 58 health providers during the 26-month study period, an average of 3.8 CMEs per quarter. The number of 1-on-1 meetings and phone calls was recorded for between 9 and 20 months in each city with quarterly averages ranging between 98.1 and 199.5 and 31.8 and 145.5 for meetings and phone calls respectively (Table 2, S3 Table). There was substantial heterogeneity in the cost of CMEs and respective unit cost per attendee as the effort expended to organize a CME was decoupled from the resulting attendance.

**Table 2:** Summary of outreach efforts and costs by city.

City	Types of outreach activity							Cost Summary	
	Phone call^§^		1-on-1 Meeting^◊^		CME^&^				
	$0.16 ($0.12, $0.20) per call		$7.14 ($5.92, $9.23) per meeting		$7.88 ($6.03, $9.86) per attendee			Avg. total cost/Qtr.	Overall Avg. Referral Cost/ Patient
	Avg. #/ Quarter	Avg. Cost/ Quarter	Avg. #/ Quarter	Avg. Cost/ Quarter	# of CMEs/ Quarter	Avg. # of Attendees	Avg. Cost/ Quarter		
**New Delhi**	145.5	$23.28 ($17.46, $29.10)	110.7	$790.68 ($655.04, $1022.29)	0.57	72	$323.40 ($247.47, $404.65)	$1,137.36 (range)	$0.69
**Kolkata**	31.8	$5.09 ($3.82, $6.36)	128.1	$914.78 ($677.75, $1,182.55)	1.05	54	$446.80 ($341.90, $559.06)	$1,366.67 (range)	$2.11
**Chennai**	132.9	$21.26 ($15.95, $26.58)	199.5	$1,424.43 ($1,055.36, $1,841.39)	1.38	38	$413.23 ($316.21, $517.06)	$1,858.92 (range)	$2.55
**Hyderabad**	108	$17.28 ($12.96, $21.60)	98.1	$700.43 ($580.75, $905.46)	0.81	66	$421.26 ($322.36, $527.12)	$1,138.97 (range)	$0.63

Average number of referrals per quarter ranged from 825 to 2065 across the four cities. Overall average referral cost per patient is the average of the per quarter per patient referral costs. Length of recordkeeping for outreach activities varies. Monthly averages were used to estimate outreach spending for the course of the experiment. Values in parenthesis indicate low and high estimates. Minor discrepancies between cost breakdown and activity sums are due to rounding.

§ Phone call cost includes $0.13 staff costs and $0.02 outreach.

◊ Meeting costs include $3.23 staff costs, $2.99 transport costs and $0.93 overhead costs.

& Workshop costs per attendee include $0.77 staff costs, $6.08 misc. costs including transport and $1.03 overhead costs.

**Fig 3:**
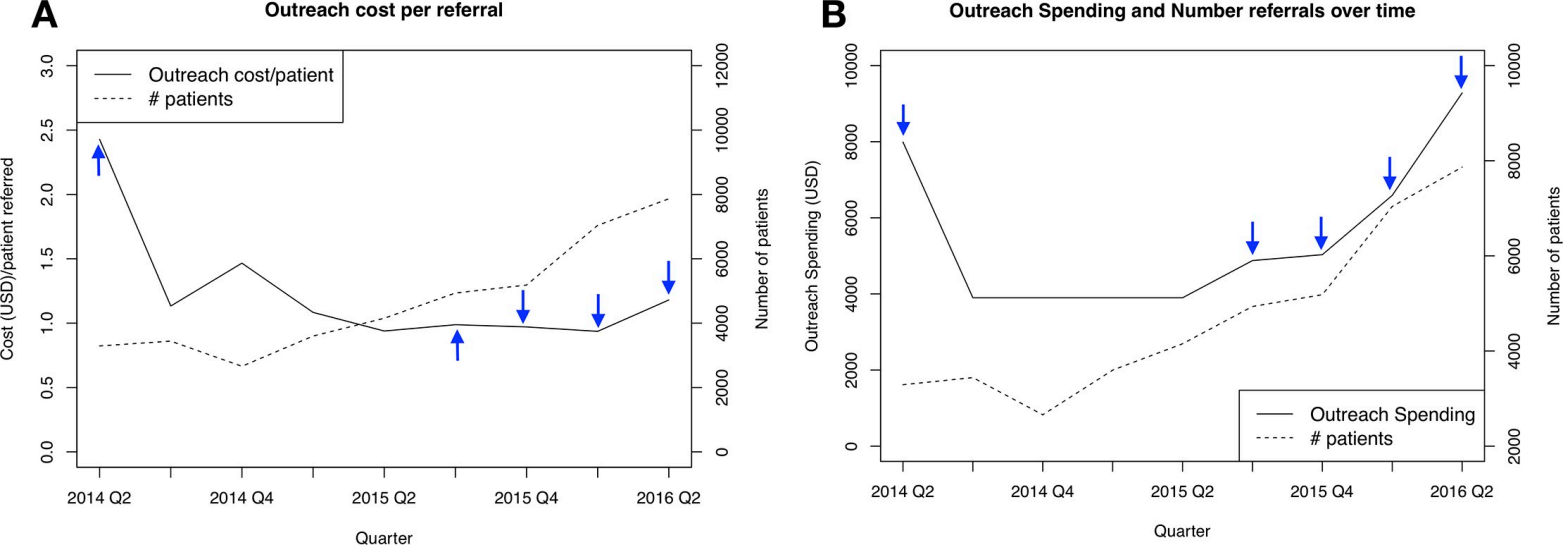
Trends in outreach spending and number of successful patient referral for Xpert testing. Fig 3A represents the trend of outreach spending cost per patient (solid line) referred plotted against the total number of patients referred (dotted line). Fig 3B plots the combined total outreach spending (solid line) against the number of patients referred (dotted line) for each quarter of the study period. Blue arrows indicate quarters where at least one CME was conducted.

In general, the combined outreach cost per Xpert testing referral generated decreased with time stabilizing at an average cost of $1.10 (Fig 3A). This ratio increased slightly in the final quarter as outreach efforts increased ahead of the project transition (Fig 3B). However, cost per referral was highly heterogeneous across the study sites, due to heterogeneous levels of demand for Xpert testing across the cities (Table 2, S1 Fig).

## Discussion

The FIND/RNTCP pediatric Xpert project demonstrated the importance and feasibility of providing a upfront Xpert testing for pediatric presumptive TB patients in four major Indian cities. Through this program, 92.6% of patients received results within 24 hours of sample receipt at the laboratories providing rapid diagnosis and drug sensitivity testing [3]. More than 42,200 children were tested yielding 3,340 cases, including 295 cases of rifampicin-resistant TB. Subsequently, in this paper, we provide important insights and empiric evidence in respect to the costs and cost dynamics associated with the efforts in promoting and improving the utility of the Xpert test in India for diagnosis pediatric TB, which are critical in formulating future policies and guidelines for Xpert testing referral efforts and coping with increased demand for Xpert testing.

Our study demonstrated a clear empiric relationship between the demand generating outreach and promotion activities and the laboratory operational adjustments needed to adequately supply of Xpert testing capacities to cope with the increasing demand for Xpert testing at all study locations. For outreach activities, we observed significant upfront costs associated with initial recruitment efforts, where cost stabilized around $1 per patient referred as the program increased its operational efficiency. As shown in Fig 3, the momentum towards program efficiency, measured as the number of patient referrals, was gained after one full year since the program initiation and directly corresponded to increasing frequency and spending on the combined outreach activities. This implies the importance of sustained periodic programming to ensure prolonged impact on health providers’ awareness and use of the Xpert test for the diagnosis of pediatric TB.

On the laboratory operational side, Xpert testing capacity (under- or overuse) was a major cost driver for average Xpert per-test cost. Laboratories with excess capacity had higher average per-test costs whereas those that met or exceeded their standard workday capacity had lower average per-test costs. However, during periods of high demand, some laboratories required a substantial amount of overtime to meet demand for quick turnaround testing. In New Delhi, a significant proportion (13.9%) of the total expenditure on testing came from testing performed in excess of the standard workday capacity, particularly at a later stage of the study when the GeneXpert referrals rapidly increased immediately following CMEs. Likewise, as the outreach efforts to promote pediatric (and potentially general TB patients Xpert testing are scaled to other cities in India, evidence on projecting demand for Xpert testing in the target area should carefully be studied and be reflected in laboratory capacity and operational adjustments.

Methodologically, our cost analysis work builds upon earlier TB diagnostic costing studies [11–13] and incorporates a laboratory’s key operational characteristics to appraise the unit cost of Xpert test unique to each laboratory in a two-step approach– 1) establish reference per-test costs based on bottom-up micro-costing methods using TAM data and 2) calculate weighted-average per-test costs based on each laboratory’s observed daily workloads. As such, the difference between the reference per-test cost for a 16-batch daily workload ($13.74) and the weighted-average per-test costs ($14.71) in New Delhi laboratory, for example, reflects the magnitude and cost associated with inefficiencies in laboratory operations (largely as a cost associated with under or over utilization of GX4 and human resource capacities). Subsequently, this measure could be used as an important indicator that measures the real-world efficiencies gaps in laboratory operations allowing programs to adjust staffing and operating hours to maximize testing efficiency.

There are several limitations in our work that may limit the interpretation and generalizability of our study findings. First, actual TAM data and parts of costing data were not directly assessed from the study laboratories due to logistical challenges and data availability issues. However, as Xpert testing procedure minimizes direct hands-on efforts of a laboratory personnel with a fixed overall test run time and use of a standardized test kit, variations in inter-operator and laboratory variability in the overall procedure and direct staff times as well as use of key laboratory consumables are likely not the major cost drivers of the Xpert test. As such, we addressed this limitation by calculating weighted per-test cost at each laboratory that reflects differences in operational conditions due to demand and capacity.

Second, unlike Xpert test costs, the unit cost of outreach spending was assessed based solely on the top-down method due to lack of adequate TAM and costing data. As transportation cost records were not kept, we had to assume a standard travel distance for relevant activities. This prevented us from directly exploring and quantifying the drivers of costs associated with inefficiencies in outreach spending as we did with the Xpert costs. Furthermore, a control group of health providers who were not reached through outreach activities was not available. Thus, we cannot directly assess the efficacy of each type of outreach activity or their combined effect relative to no direct outreach activity. However, using cost per successful patient referral for Xpert as an indicator of program efficiency, we were able to 1) demonstrate gain in program’s efficiency in generating successful Xpert referrals (Fig 3) and key cost driving components of the outreach efforts across the four study sites (Table 2).

Additionally, the variability of operations and costs observed at the four study sites may not adequately represent potential heterogeneities in costs and operations associated with the scale-up of this program in other areas in India. However, we provide a data and analytic framework that can be adopted by the on-going program expansion (or similar types of public health program implementation and scale-up) led by the RNTCP in other regions of India. Likewise, our study estimates provide an important baseline for on-going and future program scale-up operations to as a point of comparison for the outreach program and Xpert testing costs.

Our study offers a novel method to incorporate operational factors in assessing the cost of laboratory diagnostics using TAM and dynamic workload data unique to each laboratory. Given India’s plan for universal access to drug-susceptibility testing and expanded pediatric Xpert testing, our findings can serve as important evidence in efficiently balancing Xpert testing capacity and demand in areas where pediatric Xpert testing referrals may be expanded.

## Supporting information

S1 Table Breakdown of Xpert per-test unit costs. (DOCX)

S2 Table Summary of overtime days at each study laboratory according to the days of the week. (DOCX)

S3 Table Number of one-on-one meetings and phone calls recorded during observation period by city. (DOCX)

S4 Table Summary of average per attendee cost for CMEs. (DOCX)

S1 Fig Example calculation of average workload-weighted Xpert per-test unit cost. (TIFF)

S1 Calculation Example calculation of average workload-weighted Xpert per-test unit cost. (DOCX)

S1 File Costing sheet for Xpert per-test unit cost. (XLSX)

S2 File Dataset of daily lab workloads. (CSV)
