# Supplementary material for: Cost and operational impact of promoting upfront GeneXpert MTB/RIF test referrals for presumptive pediatric tuberculosis patients in India
Source: PLoS One. 2019 Apr 1;14(4):e0214675. doi: 10.1371/journal.pone.0214675 (PMC6443160; doi:10.1371/journal.pone.0214675)
Supplement: S1 Table — (DOCX) [file pone.0214675.s001.docx]

| DELHI | | | | | | | | |
| --- | --- | --- | --- | --- | --- | --- | --- | --- |
| Resource type | **Batch size** | | | | | | | |
|  | **1** | **2** | **4** | **6** | **7** | **10** | **14** | **16** |
| Overhead | 1.29 | 1.29 | 1.41 | 2.70 | 3.99 | 4.11 | 5.52 | 5.65 |
| Building Space | 0.00 | 0.00 | 0.00 | 0.00 | 0.00 | 0.00 | 0.01 | 0.01 |
| Equipment | 16.37 | 16.40 | 16.41 | 16.76 | 17.08 | 17.12 | 17.48 | 17.50 |
| Staff | 0.69 | 0.69 | 0.76 | 1.45 | 2.14 | 2.21 | 2.97 | 3.04 |
| Reagents and Chemicals | 11.98 | 23.96 | 47.92 | 71.88 | 83.86 | 119.80 | 167.72 | 191.68 |
| Consumables | 1.81 | 1.91 | 1.91 | 1.91 | 1.91 | 1.91 | 1.91 | 1.91 |
| TOTAL | 32.14 | 44.24 | 68.41 | 94.70 | 108.98 | 145.15 | 195.60 | 219.77 |
| Per 1 specimen | 32.14 | 22.12 | 17.10 | 15.78 | 15.57 | 14.51 | 13.97 | 13.74 |

| HyDERABAD | | | | | | | | |
| --- | --- | --- | --- | --- | --- | --- | --- | --- |
| Resource type | **Batch size** | | | | | | | |
|  | **1** | **2** | **4** | **6** | **7** | **10** | **14** | **16** |
| Overhead | 2.26 | 2.26 | 1.49 | 2.70 | 3.99 | 4.11 | 5.52 | 5.65 |
| Building Space | 0.00 | 0.00 | 0.00 | 0.00 | 0.00 | 0.00 | 0.01 | 0.01 |
| Equipment | 13.31 | 13.33 | 13.34 | 16.76 | 17.08 | 17.12 | 17.48 | 17.50 |
| Staff | 0.56 | 0.56 | 0.62 | 1.45 | 2.14 | 2.21 | 2.97 | 3.04 |
| Reagents and Chemicals | 11.98 | 23.96 | 47.92 | 71.88 | 83.86 | 119.80 | 167.72 | 191.68 |
| Consumables | 2.74 | 2.81 | 2.81 | 1.91 | 1.91 | 1.91 | 1.91 | 1.91 |
| TOTAL | 30.85 | 42.92 | 66.18 | 94.70 | 108.98 | 145.15 | 195.60 | 219.77 |
| Per 1 specimen | 30.85 | 21.46 | 16.55 | 15.78 | 15.57 | 14.51 | 13.97 | 13.74 |

| KOLKATA | | | | | | | | |
| --- | --- | --- | --- | --- | --- | --- | --- | --- |
| Resource type | **Batch size** | | | | | | | |
|  | **1** | **2** | **4** | **6** | **7** | **10** | **14** | **16** |
| Overhead | 0.98 | 0.98 | 1.07 | 2.05 | 3.03 | 3.13 | 4.20 | 4.30 |
| Building Space | 0.00 | 0.00 | 0.00 | 0.00 | 0.00 | 0.00 | 0.00 | 0.00 |
| Equipment | 15.29 | 15.31 | 15.32 | 15.60 | 15.86 | 15.89 | 16.19 | 16.20 |
| Staff | 0.56 | 0.56 | 0.62 | 1.18 | 1.74 | 1.79 | 2.41 | 2.47 |
| Reagents and Chemicals | 11.98 | 23.96 | 47.92 | 71.88 | 83.86 | 119.80 | 167.72 | 191.68 |
| Consumables | 1.42 | 1.47 | 1.47 | 1.47 | 1.47 | 1.47 | 1.47 | 2.20 |
| TOTAL | 30.23 | 42.28 | 66.40 | 92.18 | 105.96 | 142.09 | 191.99 | 216.85 |
| Per 1 specimen | 30.23 | 21.14 | 16.60 | 15.36 | 15.14 | 14.21 | 13.71 | 13.55 |

**S1 Table.** Breakdown of per-test cost of Xpert by resource type and size of sample batch at each study laboratory
