# Supplementary material for: Cost and operational impact of promoting upfront GeneXpert MTB/RIF test referrals for presumptive pediatric tuberculosis patients in India
Source: PLoS One. 2019 Apr 1;14(4):e0214675. doi: 10.1371/journal.pone.0214675 (PMC6443160; doi:10.1371/journal.pone.0214675)
Supplement: S2 Table — (DOCX) [file pone.0214675.s002.docx]

| **Delhi** | **Mon.** | **Tues.** | **Wed.** | **Thu.** | **Fri.** | **Sat.** | **Sun** |
| --- | --- | --- | --- | --- | --- | --- | --- |
| Days over | 40 | 34 | 33 | 26 | 26 | 14 | 6 |
| Days in exp. | 117 | 117 | 118 | 118 | 117 | 117 | 117 |
| Prop. (%) | 34.2 | 29.0 | 28.0 | 22.0 | 22.2 | 12.0 | 5.1 |

| **Kolkata** | **Mon.** | **Tues.** | **Wed.** | **Thu.** | **Fri.** | **Sat.** | **Sun** |
| --- | --- | --- | --- | --- | --- | --- | --- |
| Days over | 2 | 6 | 5 | 6 | 5 | 4 | 9 |
| Days in exp. | 117 | 117 | 118 | 118 | 117 | 117 | 117 |
| Prop. (%) | 1.8 | 5.1 | 4.2 | 5.1 | 4.3 | 3.4 | 7.7 |

| **Hyderabad** | **Mon.** | **Tues.** | **Wed.** | **Thu.** | **Fri.** | **Sat.** | **Sun** |
| --- | --- | --- | --- | --- | --- | --- | --- |
| Days over | 1 | 10 | 18 | 15 | 14 | 12 | 44 |
| Days in exp. | 117 | 117 | 118 | 118 | 117 | 117 | 117 |
| Prop. (%) | 1.0 | 8.5 | 15.3 | 12.7 | 12.0 | 10.2 | 37.6 |

| **Chennai** | **Mon.** | **Tues.** | **Wed.** | **Thu.** | **Fri.** | **Sat.** | **Sun** |
| --- | --- | --- | --- | --- | --- | --- | --- |
| Days over | 7 | 7 | 3 | 7 | 4 | 1 | 0 |
| Days in exp. | 117 | 117 | 118 | 118 | 117 | 117 | 117 |
| Prop. (%) | 6.0 | 5.9 | 2.5 | 5.9 | 3.4 | 1.0 | 0.0 |

**Table S2.** Summary of overtime days at each study laboratory according to the days of the week
