# Supplementary material for: Cost and operational impact of promoting upfront GeneXpert MTB/RIF test referrals for presumptive pediatric tuberculosis patients in India
Source: PLoS One. 2019 Apr 1;14(4):e0214675. doi: 10.1371/journal.pone.0214675 (PMC6443160; doi:10.1371/journal.pone.0214675)
Supplement: S3 Table — (DOCX) [file pone.0214675.s003.docx]

| **Delhi** | **Category** | **Number recorded** | **Period recorded (months)** | **Avg. number/quarter** |
| --- | --- | --- | --- | --- |
|  | 1-on-1 meeting | 369 | 10 | 110.7 |
|  | Phone call | 485 | 10 | 145.5 |
|  |  |  |  |  |
| **Kolkata** | **Category** | **Number recorded** | **Period recorded (months)** | **Avg. number/month** |
|  | 1-on-1 meeting | 384 | 9 | 128 |
|  | Phone call | 95 | 9 | 31.7 |
|  |  |  |  |  |
| **Chennai** | **Category** | **Number recorded** | **Period recorded (months)** | **Avg. number/month** |
|  | 1-on-1 meeting | 798 | 12 | 199.5 |
|  | Phone call | 532 | 12 | 133 |
|  |  |  |  |  |
| **Hyderabad** | **Category** | **Number recorded** | **Period recorded (months)** | **Avg. number/month** |
|  | 1-on-1 meeting | 654 | 20 | 98.1 |
|  | Phone call | 720 | 20 | 108 |

**Table S3.** Number of 1-on-1 meetings and phone calls recorded during observation period by city
