# Supplementary material for: Cost and operational impact of promoting upfront GeneXpert MTB/RIF test referrals for presumptive pediatric tuberculosis patients in India
Source: PLoS One. 2019 Apr 1;14(4):e0214675. doi: 10.1371/journal.pone.0214675 (PMC6443160; doi:10.1371/journal.pone.0214675)
Supplement: S4 Table — (DOCX) [file pone.0214675.s004.docx]

| **Average per attendee cost for CMEs** | | USD |
| --- | --- | --- |
| Observed conditions | Cost/attendee, weighted by CME | 7.88 |
| Low cost conditions | Cost/attendee, weighted by CME | 6.03 |
| High cost conditions | Cost/attendee, weighted by CME | 9.86 |

**Table S4, Summary of average per attendee cost for CMEs**
