# Supplementary figures and images for: Cost and operational impact of promoting upfront GeneXpert MTB/RIF test referrals for presumptive pediatric tuberculosis patients in India
Source: PLoS One. 2019 Apr 1;14(4):e0214675. doi: 10.1371/journal.pone.0214675 (PMC6443160; doi:10.1371/journal.pone.0214675)

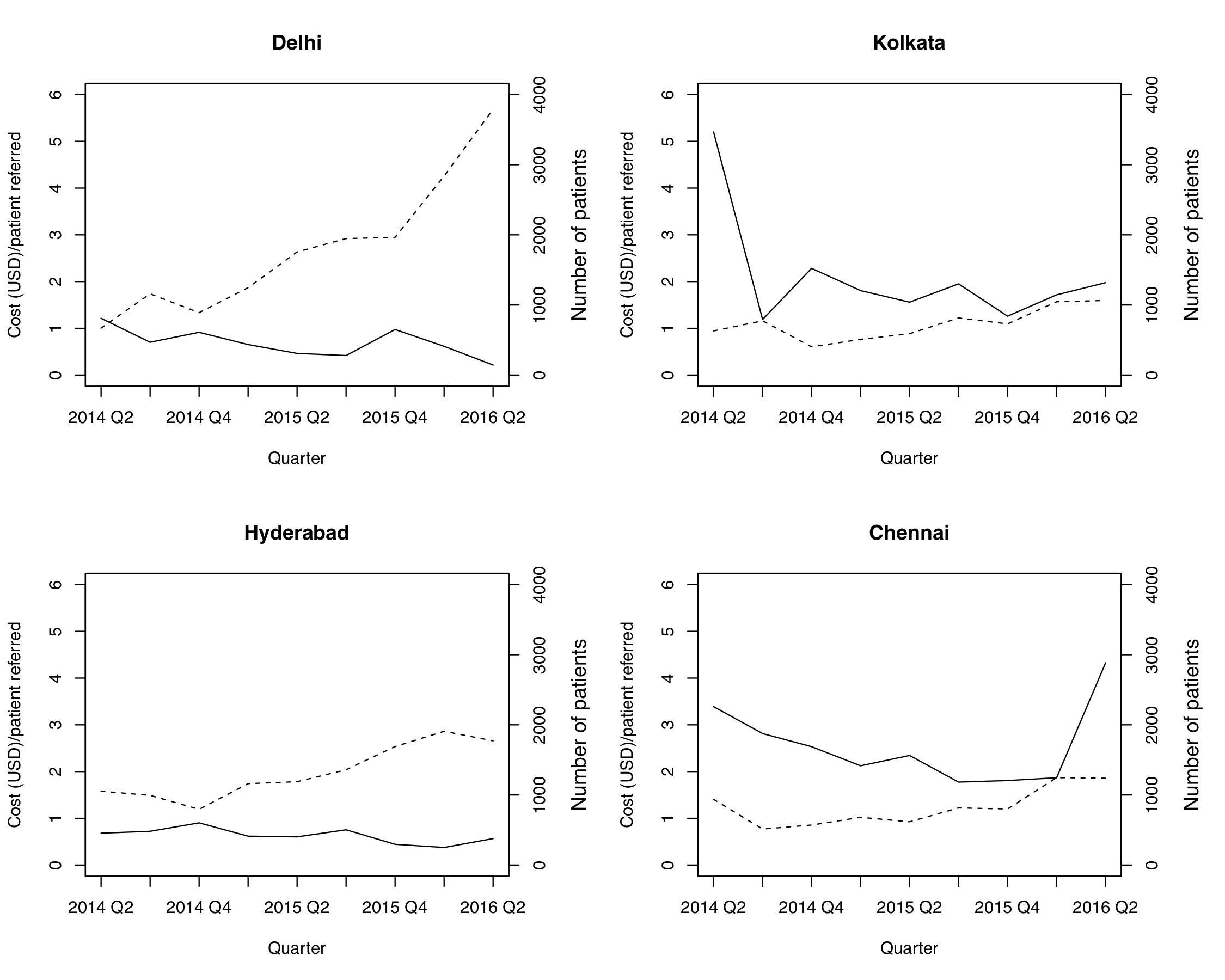

Supplement: S1 Fig — (TIFF) [file pone.0214675.s005.tiff]
