# Supplementary material for: Cost and operational impact of promoting upfront GeneXpert MTB/RIF test referrals for presumptive pediatric tuberculosis patients in India
Source: PLoS One. 2019 Apr 1;14(4):e0214675. doi: 10.1371/journal.pone.0214675 (PMC6443160; doi:10.1371/journal.pone.0214675)
Supplement: S1 Calculation — (DOCX) [file pone.0214675.s006.docx]

**Example calculation of average workload-weighted Xpert per-test unit cost.**

For a lab, with one Xpert machine (16 test capacity during standard working hours). On Day 1, 13 Xpert tests are performed. On Day 2, 20 Xpert tests are performed.

**Day 1 workload-weighted cost:**

Total cost for day=10*(10 batch unit cost)+2*(2 batch cost)+1*(1 batch cost)+3*(penalty for unused capacity)

=10*($18.53)+2*($25.69)+1*($35.19)+3*($0.85)= $274.42

Average workload weighted cost for the day=$274.42/13=**$21.11**

**Day 2 workload-weighted cost:**

Total cost for day=16*(16 batch unit cost)+4*(4 batch unit cost)

=16*($17.79)+4*($20.87)=$ 368.12

Average workload weighted cost for the day=$368.12/20=**$18.41**

**Average workload-weighted Xpert per-test unit cost for Day 1 and Day 2:**

Average($21.11,$18,41) = **$19.76**
